# Transcriptomic Analysis of Ovaries from Pigs with High And Low Litter Size

**DOI:** 10.1371/journal.pone.0139514

**Published:** 2015-10-01

**Authors:** Xiaodong Zhang, Long Huang, Tao Wu, Yifang Feng, Yueyun Ding, Pengfei Ye, Zongjun Yin

**Affiliations:** Key Laboratory of Local Animal Genetic Resources Conservation and Bio-breeding of Anhui province, College of Animal Science and Technology, Anhui Agricultural University, Hefei, Anhui Province, People’s Republic of China; University of Lleida, SPAIN

## Abstract

Litter size is one of the most important economic traits for pig production as it is directly related to the production efficiency. Litter size is affected by interactions between multiple genes and the environment. While recent studies have identified some genes associated with prolificacy in pigs, transcriptomic studies of specific genes affecting litter size in porcine ovaries are rare. In order to identify candidate genes associated with litter size in swine, we assessed gene expression differences between the ovaries of Yorkshire pigs with extremely high and low litter sizes using the RNA-Seq method. A total of 1 243 differentially expressed genes were identified: 897 genes were upregulated and 346 genes were downregulated in high litter size ovary samples compared with low litter size ovary samples. A large number of these genes related to steroid hormone regulation in animal ovaries, including 59 Gene Ontology terms and 27 Kyoto Encyclopedia of Genes and Genomes pathways involved in steroid biosynthesis and ovarian steroidogenesis. From these differentially expressed genes, we identified a total of 11 genes using a bioinformatics screen that may be associated with high litter size in Yorkshire pigs. These results provide a list of new candidate genes for porcine litter size and prolificacy to be further investigated.

## Introduction

Litter size is one of the most important economic factors in pig production and is affected by interactions between multiple genes and the environment [1, 2]. The size of litters varies between pigs in different breeding farms. Altering litter size by conventional breeding methods can be slow, and has shown low heritability. However, using marker assisted selection (MAS) can speed up genetic improvements in litter size traits [3]. As the ovary directly mediates ovulation, it has a significant impact on the fecundity of mammals, and therefore, genetic differences in the ovaries may contribute to the observed differences in litter size [4, 5].

There has been some recent progress in characterizing the major genes involved in the prolificacy of swine, such as the estrogen receptor (*ESR*) [6, 7], follicle-stimulating hormone beta subunit (*FSHB*) [8], retinol-binding protein 4 (*RBP4*) [9, 10], prolactin receptor (*PRLR*) [9], melatonin receptor 1a (*MTNR1A*) [11], osteopontin (*OPN*) [12], bone morphogenetic protein (*BMP*) families [13], and growth differentiation factor (*GDF9*) [14]. With the recent publication of the pig genome, more candidate genes or quantitative trait loci (QTLs) have been extensively investigated for their involvement in porcine litter size [3, 15, 16]; but genomic location, function and interaction of these genes requires further research.

RNA sequencing (RNA-Seq) can measure genes, both quantitatively and functionally, at the transcriptome level [17]. To date, RNA-Seq has been used to study specific ovarian genes of important livestock animals (e.g., yak [18], and goat [19, 20]), but transcriptomic studies of specific genes in pigs are rare, and have mainly focused on the molecular regulation mechanism of fat deposition and muscle development [21–23]. Therefore, transcriptomic analysis of pig ovaries using RNA-Seq may explain heredity of porcine fecundity, and be used to identify key genes relating to litter size.

Yorkshire is one of the world's most famous lean pig breeds, and is known for its higher fertility and high litter size [24, 25]. However, in pig production, some Yorkshire pigs always have low litter sizes, which seriously restricts productivity. In the present study, we sampled ovaries of Yorkshire pigs with extremely high and low litter sizes, and analyzed the differential expression of genes (DEGs) using RNA-Seq. The aim of this analysis was to identify major genes that control prolificacy, and thus provide a molecular basis for genetic improvement of reproductive traits in swine.

## Materials and Methods

### Ethics statement

Yorkshire (a Danish lean-type breed) pigs were obtained from the Anhui Daziran Primary Pig Breeding Farm, Huaibei, Anhui, China. All experimental procedures and sample collection were performed according to the Regulations for the Administration of Affairs Concerning Experimental Animals (Ministry of Science and Technology, China; revised in June 2004) and approved by the Institutional Animal Care and Use Committee of Anhui Agricultural University, Hefei, China, under permit No. ZXD-P20140809. This report fully adhered to the ARRIVE Guidelines for the reporting of animal research [26]. A completed ARRIVE guideline checklist is included (S1 Checklist). The animals were reared in the same environment and fed the same diet *ad libitum* during the experimental period. Food was withheld from the animals on the night before they were slaughtered.

### Animals and ovary collection

A total of twelve healthy female pigs were used in this study from two groups: the extreme high litter size group (YH: n = 6), and the extreme low litter size group (YL: n = 6) (Table 1), representing pigs with high and low fecundity, respectively. In order to reduce, as far as is possible, the effects of age and parity on litter size, three pigs of similar age and parity from each group were selected as biological replicates for RNA-Seq. Their intact ovaries were rapidly harvested from their carcasses and immediately frozen in liquid nitrogen. All tissue samples were stored at −80°C until the total RNA extraction procedure was performed.

**Table 1:** Prolificacy characteristics of Yorkshire pigs with high and low litter sizes.

Group	Sample ID	Months of age	Total parity numbers	TNB	NBA	Use
YH	YH1	30	4	16.5 ± 1.8	13.0 ± 0.4	RNA-Seq and qPCR
	YH2	30	4	16.5 ± 1.8	13.0 ± 0.4	RNA-Seq and qPCR
	YH3	35	5	17.0 ± 1.1	15.2 ± 1.3	RNA-Seq and qPCR
	YH4	45	7	14.6 ± 1.8	11.0 ± 1.2	qPCR
	YH5	46	7	16.4 ± 1.4	13.6 ± 1.0	qPCR
	YH6	45	7	14.6 ± 1.8	11.0 ± 1.2	qPCR
YL	YL1	30	4	5.2 ± 1.9	5.2 ± 1.9	RNA-Seq and qPCR
	YL2	30	4	5.0 ± 0.7	4.0 ± 0.4	RNA-Seq and qPCR
	YL3	35	5	5.8 ± 2.1	5.0 ± 1.6	RNA-Seq and qPCR
	YL4	30	4	6.3 ± 2.1	5.3 ± 1.4	qPCR
	YL5	31	4	5.5 ± 1.7	4.6 ± 0.6	qPCR
	YL6	34	5	5.3 ± 0.8	4.5 ± 0.9	qPCR

Values are means ± standard error. YH represents the extreme high litter size group, and YL represents the extreme low litter size group. TNB represents the total number born, and NBA represents the number born alive.

### mRNA library preparation and sequencing

The ovaries were completely ground and total RNA was extracted using TRIzol (Invitrogen, Carlsbad, CA, USA). The quality of the total RNA was checked using the Agilent 2100 Bioanalyzer system (Santa Clara, CA, USA). A total amount of 3 μg RNA per sample was used as input material for the RNA sample preparations. Sequencing libraries were generated using NEBNext® Ultra^TM^ RNA Library Prep Kit for Illumina® (NEB, USA) following manufacturer’s recommendations. Briefly, mRNA was extracted from total RNA using oligo (dT) magnetic beads and sheared into short fragments of about 200 bases. These fragmented mRNAs were then used as templates for cDNA synthesis. The cDNAs were then PCR amplified to complete the library. The cDNA library was sequenced using an Illumina HiSeq^TM^ 2000 platform.

### Analysis of RNA-Seq data

Raw RNA-Seq reads were processed through in-house perl scripts. Clean reads were obtained by removing reads containing low quality reads and/or adaptor sequences from raw reads [27], and mapped to the pig genome (*Sus scrofa* 10.2) using TopHat software [28], allowing up to two base mismatches. The gene expression level was then calculated using the reads per kilo bases per million reads (RPKM) method [29]. We considered the gene was expressed exclusively in one of the two groups if its RPKM ≥ 0.3 in one group and < 0.3 in the other group. Differential expression analysis was performed using the Benjamini and Hochberg’s approach for controlling the false discovery rate [30]. Genes with an adjusted *P* value < 0.05 were assigned as differentially expressed (DEG).

DEG lists were submitted to the databases of Gene Ontology (GO) and Kyoto Encyclopedia of Genes and Genomes (KEGG) for enrichment analysis of the significant overrepresentation of GO terms and KEGG-pathway categories [31, 32]. In all tests, *P* values were calculated using the Benjamini-corrected modified Fisher’s exact test and ≤ 0.05 was taken as a threshold of significance.

### Quantitative PCR analysis

The RNA-Seq results were validated using an RNA samples from the YH group (n = 6) and the YL group (n = 6), respectively. Six DEGs enriched in the ovarian steroidogenesis pathway, two DEGs (one upregulated, and the other downregulated in the YH group) were enriched for metabolic pathways, two DEGs with the highest numbers of reads in both YH and YL, and two genes (one expressed only in the YH group, and the other expressed only in the YL group) were analyzed by quantitative PCR (qPCR) (S1 Table). Total RNA (1.0 mg) was used to synthesize first-strand cDNA using PrimeScript RT Master Mix (TaKaRa, Osaka, Japan). Q-PCR was performed using SYBR Premix Ex Taq (TaKaRa, Osaka, Japan) in the CFX96 Real-Time PCR Detection System (Bio-Rad, Hercules, CA, USA). The primers were designed using the Primer Express (Applied Biosystems) software. All the mRNA nucleotide sequences were obtained from the NCBI Entrez Nucleotide database (http://www.ncbi.nlm.nih.gov/sites/entrez?db=nuccore&itool=toolbar), or from the EMBL-EBI database (http://www.ebi.ac.uk). The primers used for qPCR are listed in S1 Table. The thermal cycling conditions were 95°C for 10 min, followed by 40 cycles of 95°C for 15 s and 60°C for 1 min. There were three replicates for each amplification. Cycle threshold (Ct) values were analysed using a linear mixed model as described below.

$$Ct_{jkl}=\;\mu \;+L_{j}+\;A_{k}+\;P_{l}+\;e_{jkl};$$

in which L is the effect of fecundity, A is the fixed effect of age, and P is the fixed effect of parity of animal [33]. Then the Ct values were transformed to quantities using the comparative Ct method. Data was normalized using the porcine β-actin reference gene. Comparison of gene expression levels conditional on prolificacy levels was performed using the *t*-test, and correlations between qPCR and RNA-Seq measures were calculated.

## Results

### Overview of sequencing data

After removing the low quality and adaptor sequences, we obtained approximately 52 to 66 million (M) clean reads for six RNA-Seq libraries, and high percentages of mapped reads ranging from 79.15 to 80.68%. Most mapped reads were located within an exon (71.6 to 85.6%) while a smaller percentage of mapped reads (less than 19. 0%) were located within the introns and intergenic regions (Table 2). These results indicated that our six libraries were of high quality, and had high coverage of the pig genome. This allowed us to compare the ovary transcriptomes from pigs with high and low litter size. The data is available from the Sequence Read Archive (SRA) (Accession no. SRP058401, Bioproject: PRJNA283575).

**Table 2:** RNA sequencing results of mRNA from the ovaries of Yorkshire pigs with high and low litter sizes.

Sample ID^1^	Clean reads, M^2^	Mapping rate, %^3^	exons, %	Intron, %	Intergenic, %
YH1	52.66	80.67	82.20	3.70	14.20
YH2	60.14	80.68	85.60	2.40	12.00
YH3	64.89	80.07	82.20	4.10	13.70
YL1	65.60	79.80	73.20	8.30	18.60
YL2	57.76	80.16	71.60	9.40	19.00
YL3	65.04	79.15	78.30	6.00	15.70

^1.^ YH1, YH2, YH3 and YL1, YL2, YL3 are replicate from the YH and YL groups. YH represents the extreme high litter size group, and YL represents the extreme low litter size group.

^2.^ Indicates millions of reads.

^3.^ Uses the *Sus scrofa* 10.2 as the reference genome annotation to classify the mapping tags into the different regions. Ratio was calculated by the number of tags on each region divided by the total tags on the whole genome.

### Differentially expressed genes between YH and YL groups

**Fig 1:**
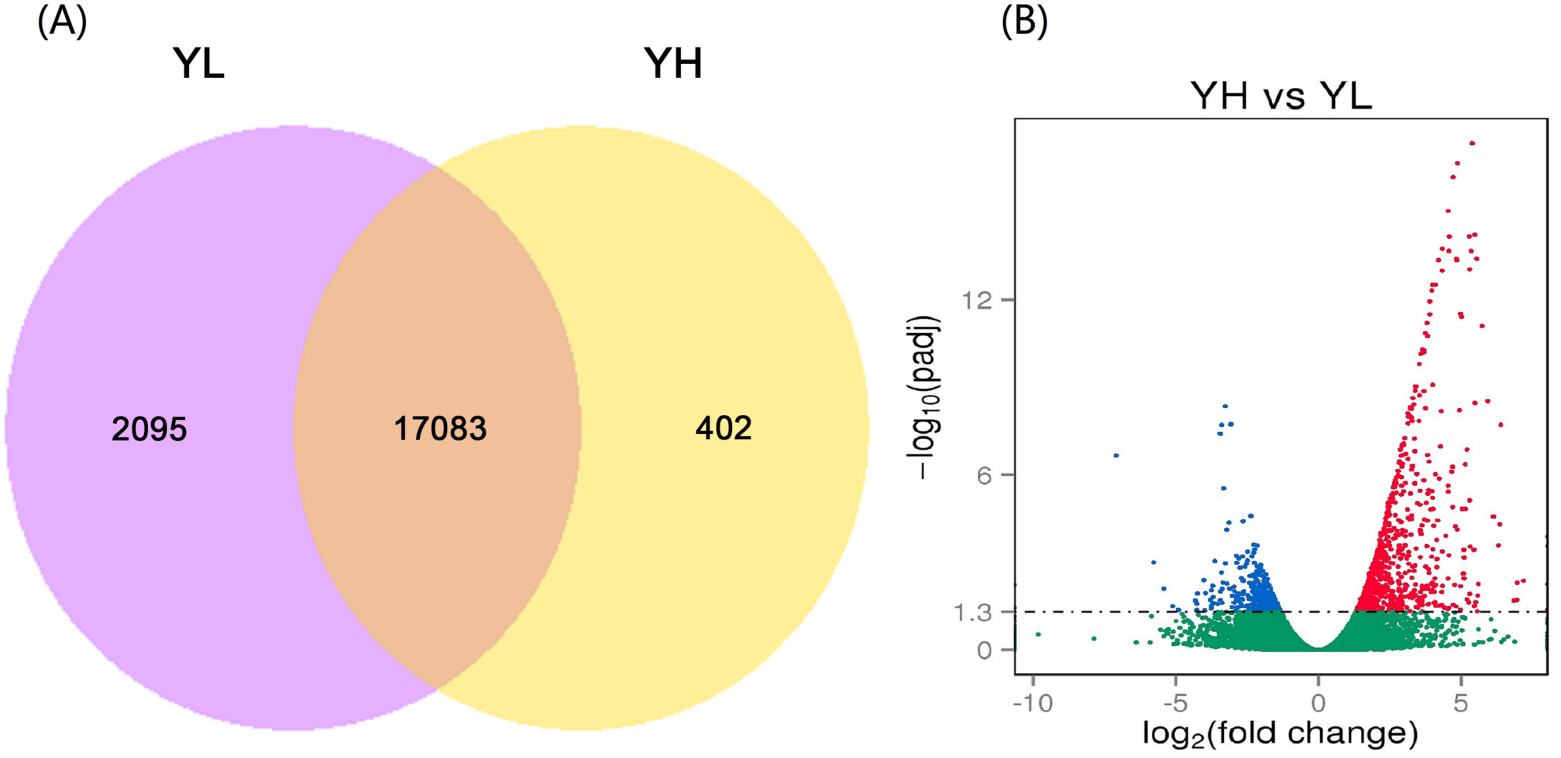
Comparative results of gene expression levels and differentially expressed gene distributions between the ovaries of Yorkshire pigs with extremely high (YH) and low (YL) litter size. (A) Venn diagram showing genes only expressed in the YH group (yellow circle), only expressed in the YL group (light red circle), and common to both groups (intersection). (B) Scatter plot of differentially expressed genes (YH vs. YL). Red points represent upregulated genes with log_2_ (fold change) > 1 and padj < 0.05 (–log10 (padj) ≥ 1.3); Blue points represent downregulated genes with log_2_ (fold change) < -1 and padj < 0.05 (–log10 (padj) ≥ 1.3). Green points represent genes with no significant difference. Fold change = gene normalized expression of the YH group / gene normalized expression of the YL group.

After mapping to the pig genome, a total of 17 485 and 19 178 genes were obtained from the YH and YL libraries, respectively. Four hundred and two genes were expressed only in YH, 2 059 genes were expressed only in YL, and 17 083 genes were co-expressed in both libraries (Fig 1A). A total of 1 243 genes were differentially expressed between the two groups, in which 897 genes were upregulated and 346 genes were downregulated in the YH group (Fig 1B and S2 Table). The 10 most differentially expressed genes (log_2_FoldChange ≥4) from the total of 1 243 DEGs identified between the high and low litter size samples were: homogentisate 1,2 dioxygenase (*HGD*); phosphoenylpyruvate carboxykinase (*PCK1*), *HSD17B2*; early growth response 4 (*EGR4*), a member of the ras oncogene family (*RAB33A*); solute carrier protein family 6 (*SLC6A20B*); zinc finger protein (*GLI1*); U6 spliceosomal RNA (*U6*); solute carrier protein family 7 (*SLC7A11*); and spectrin alpha chain eyrythrocytic 1 (*SPTA1*), respectively (Table 3).

**Table 3:** Detailed information on the top 10 most differentially expressed genes.

Gene Name	readcount_ YH	readcount_ YL	log2FoldChange	*P* val	*P* adj	Up/Down (YH/YL)	Interpro Description
*HGD*	77.54	0.53	7.18	8.29E-05	0.00436	Up	Homogentisate 1,2-dioxygenase
*PCK1*	417.45	3.35	6.96	0.000101	0.005071	Up	Phosphoenolpyruvate carboxykinase,
*HSD17B2*	235.38	1.91	6.95	0.000661	0.019735	Up	Hydroxysteroid (17-beta) dehydrogenase 2, mRNA.
*EGR4*	245.79	2.15	6.84	0.000716	0.020727	Up	Early growth response 4
*RAB33A*	488.52	5.84	6.39	4.92E-11	1.96E-08	Up	Member RAS oncogene family (RAB33A), mRNA
*SLC6A20B*	0.81	15.67	-4.27	0.000992	0.026366	Down	Solute carrier family 6, member 20B
*GLI1*	0.81	16.24	-4.32	0.000719	0.020791	Down	Zinc finger protein GLI1
*U6*	0.41	12.20	-4.91	0.002086	0.043381	Down	U6 spliceosomal RNA
*SLC7A11*	0.39	16.53	-5.42	0.000196	0.008195	Down	Solute carrier family 7, member 11
*SPTA1*	2.43	331.59	-7.09	7.43E-10	2.25E-07	Down	Spectrin, alpha, erythrocytic 1

### Functional enrichment analysis of differentially expressed genes

To define the biological functions of the 1 243 DEGs, GO and KEGG analysis were carried out. Fifty-nine significantly enriched GO terms (corrected *P* < 0.05) were identified, including single-organism metabolic process, lipid metabolic process, oxidation-reduction process, lipid biosynthetic process, organic acid metabolic process, carboxylic acid metabolic process, oxoacid metabolic process, small molecule metabolic process, fatty acid metabolic process, cellular lipid metabolic process, catalytic activity, steroid metabolic process, and glucose metabolic process (Table 4 and S3 Table). Meanwhile, 27 significantly enriched KEGG pathways were identified, including metabolic pathways, valine/leucine and isoleucine degradation, fatty acid metabolism, carbon metabolism, steroid biosynthesis, butanoate metabolism, ovarian steroidogenesis, biosynthesis of unsaturated fatty acids, PPAR signaling pathway, synaptic vesicle cycle, and biosynthesis of amino acids (Table 5 and S4 Table). Among these GO terms and KEGG pathways, the steroid biosynthesis and ovarian steroidogenesis pathways are the ones related to steroid hormone regulation in animal ovaries and therefore likely to be contributing to litter size. However, as most GO and KEGG assignments and distributions are related to reproduction, growth and development, and metabolism, our results indicate that the DEGs are involved in a wide range of regulatory functions in porcine ovaries.

**Table 4:** The top 10 most significantly enriched Gene Ontology (GO) terms from differentially expressed genes (DEGs) in Yorkshire pigs.

GO term	Description	*P* value	Corrected *P* value	DEGs
GO:0044710	single-organism metabolic process	4.91E-19	9.99E-16	221
GO:0055114	oxidation-reduction process	8.01E-15	8.15E-12	115
GO:0044281	small molecule metabolic process	1.03E-09	3.01E-07	97
GO:0006629	lipid metabolic process	3.56E-15	4.83E-12	82
GO:0006082	organic acid metabolic process	4.36E-10	1.37E-07	58
GO:0019752	carboxylic acid metabolic process	4.36E-10	1.37E-07	58
GO:0043436	oxoacid metabolic process	4.36E-10	1.37E-07	58
GO:0044255	cellular lipid metabolic process	4.83E-09	1.23E-06	54
GO:0008610	lipid biosynthetic process	3.86E-10	1.37E-07	51
GO:0006631	fatty acid metabolic process	3.07E-09	8.32E-07	22

**Table 5:** The top ten significantly enriched Kyoto Encyclopedia of Genes and Genomes pathways from differentially expressed genes (DEGs).

Pathway ID	Pathway	*P* value	Corrected *P* value	DGEs
ssc01100	Metabolic pathways	1.14E-18	2.90E-16	191
ssc01200	Carbon metabolism	2.64E-11	1.67E-09	35
ssc00280	Valine, leucine and isoleucine degradation	3.04E-12	3.86E-10	26
ssc01212	Fatty acid metabolism	1.05E-11	8.92E-10	25
ssc04913	Ovarian steroidogenesis	2.96E-08	1.08E-06	21
ssc00071	Fatty acid degradation	5.62E-07	1.59E-05	17
ssc00100	Steroid biosynthesis	2.08E-09	1.06E-07	16
ssc00650	Butanoate metabolism	5.30E-09	2.24E-07	16
ssc01040	Biosynthesis of unsaturated fatty acids	3.97E-07	1.26E-05	12
ssc00062	Fatty acid elongation	1.40E-06	3.56E-05	12

### Validation of DEGs by qPCR

**Fig 2:**
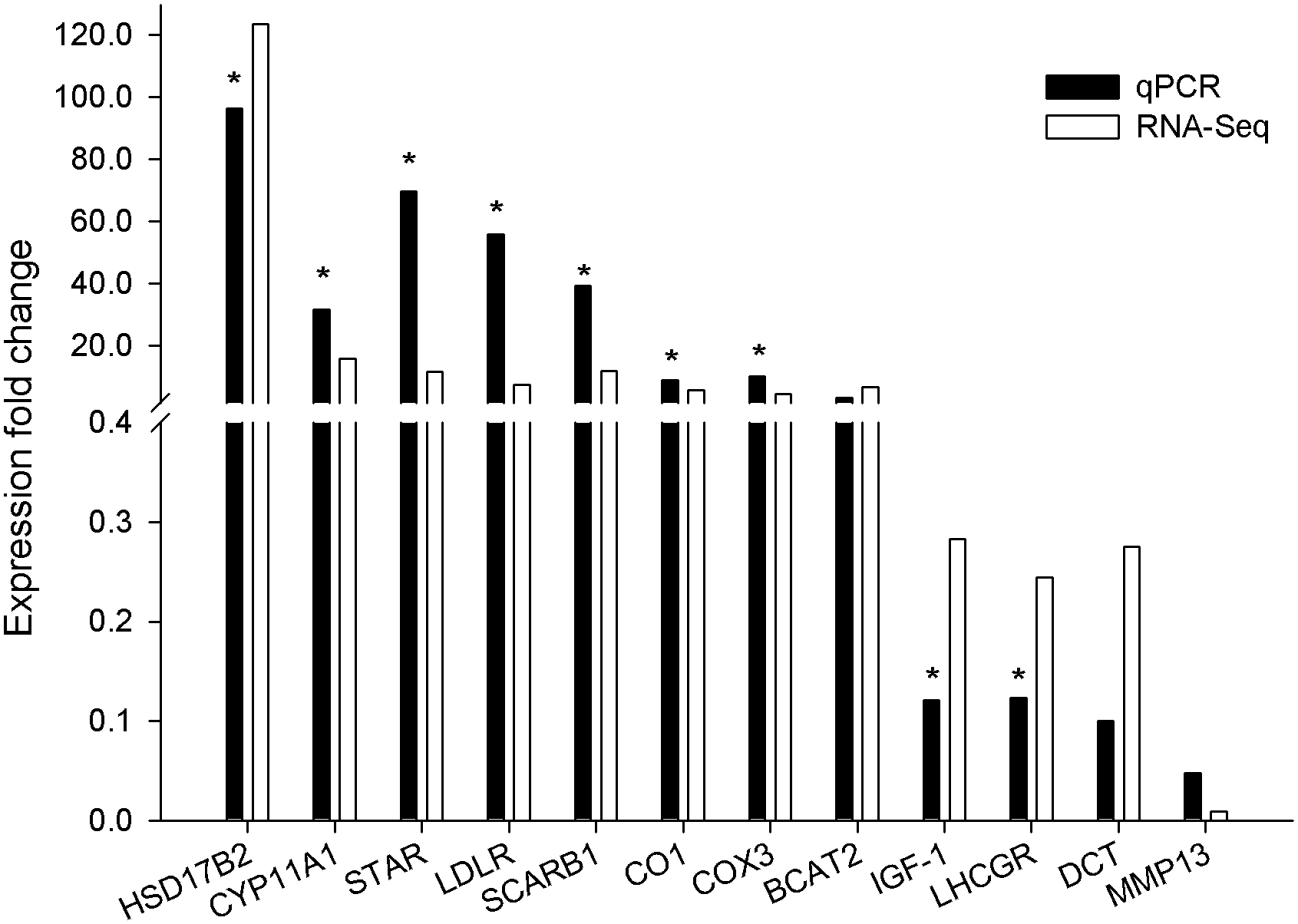
Verification of the RNA-Seq results using the qPCR method. Fold change values greater than 2 and *P* < 0.05 indicate overexpression in the YH group, and fold change values less than 0.5 and *P* < 0.05 indicate overexpression in the YL group. Genes with asterisk differ significantly (*P* < 0.05).

Twelve genes were used for qPCR analysis from the ovaries of the YH and YL groups to validate the expression profiles obtained by RNA-Seq. Consistent with the RNA-Seq findings, we verified that hydroxysteroid (17-beta) dehydrogenase 2 (HSD17B2), cytochrome P450 E class group 1 (*CYP11A1*), steroidogenic acute regulatory protein (*STAR*), low density lipoprotein receptor (*LDLR*), scavenger receptor class B member 1 (*SCARB1*), cytochrome c oxidase, subunit I domain (*CO1*), and cytochrome c oxidase subunit III domain (*COX3*) genes were all upregulated, while luteinizing hormone/choriogonadotopin receptor (*LHCGR*), and insulin-like growth factor 1 (*IGF-1*) genes were downregulated in high litter size samples. Matrix metallopeptidase 13 (*MMP13*) gene was not significant difference between the two groups. However, branched-chain amino acid aminotransferase II (*BCAT2*) and dopachrome tautomerase (*DCT*) genes were validated as different expression with *P* > 0.05 in YH versus YL groups (Table 6 and Fig 2). Therefore, results obtained from RNA-Seq were statistically confirmed for 83.3% of the tested genes by qPCR. In all cases, the relative fold change of gene expression was in the same direction between the RNA-Seq and qPCR data.

**Table 6:** Summary of qPCR and RNA-Seq results.

Gene	qPCR results				RNA-Seq Fold Change (H/L)	Significant diffs.	Confirmed	Correlation RNA-Seq vs. qPCR
	YH mean (SE)	YL mean (SE)	Fold Chang (H/L)	*P* value (H/L)				
*HSD17B2*	0.02372	0.00025	96.419	0.037	123.469	Up	Yes	0.85
*CYP11A1*	0.64853	0.02040	31.789	0.003	15.909	Up	Yes	0.83
*STAR*	1.23509	0.01774	69.642	0.008	11.766	Up	Yes	0.56
*LDLR*	0.03103	0.00056	55.888	0.001	7.555	Up	Yes	0.74
*SCARB1*	0.67635	0.01722	39.268	0.010	12.028	Up	Yes	0.62
*CO1*	3.26406	0.35572	9.176	0.005	5.799	Up	Yes	0.85
*COX3*	3.84001	0.37585	10.217	0.003	4.490	Up	Yes	0.76
*BCAT2*	0.53987	0.16381	3.296	0.293	6.860	Up	No	0.39
*IGF-1*	0.03046	0.25306	0.120	0.037	0.283	down	Yes	0.82
*LHCGR*	0.02432	0.19728	0.123	0.009	0.244	down	Yes	0.63
*DCT*	0.00006	0.00058	0.100	0.174	0.275	down	No	0.28
*MMP13*	0.00003	0.00061	0.048	0.322	0.009	No	Yes	0.65

### Identification of candidate genes for pig litter size

**Fig 3:**
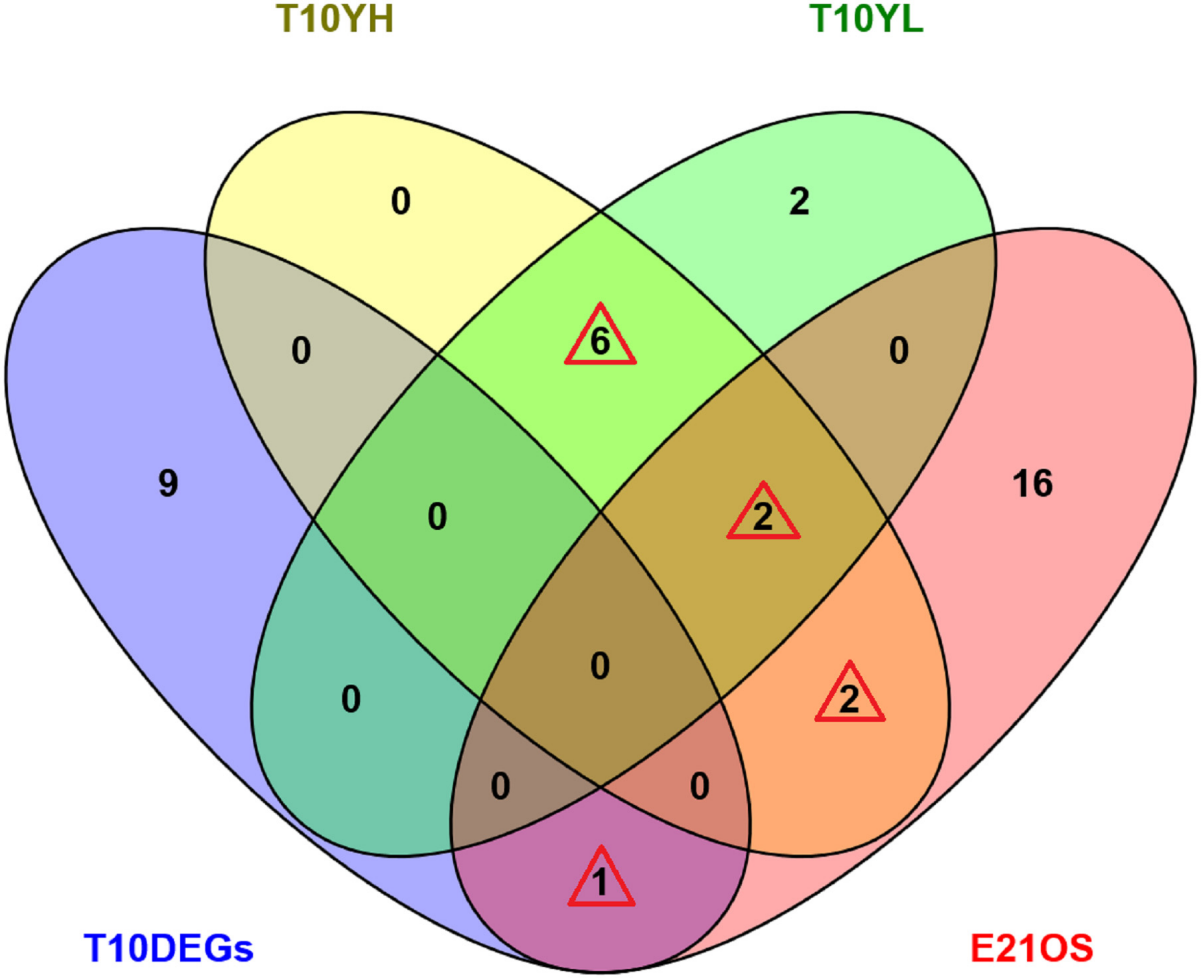
Venn diagram for identification of key genes influencing porcine litter size. T10DEGs represents the top 10 most DEGs between the high and low litter size samples; T10YH represents the top 10 most expressed genes in the high litter size samples; T10YL represents the top 10 most expressed genes in the low litter size samples; E21OS represents the 21 genes enriched in the steroid metabolic process and ovarian steroidogenesis. Six, in the light green overlapping set surrounded by the red triangle, represents the *CO1*, *GPX3*, *MSMB*, *COX3*, *TIMP1*, and *CYTB* genes; two, in the brown overlapping set surrounded by the red triangle, represents the *STAR* and *HSD3B* genes; two, in the light brown overlapping set surrounded by the red triangle, represents the *CYP11A1* and *SCARB1* genes; and one, in the violet overlapping set surrounded by the red triangle, represents the *HSD17B2* gene. Therefore, 11 genes (*CO1*, *GPX3*, *MSMB*, *COX3*, *TIMP1*, *CYTB*, *STAR*, *HSD3B*, *CYP11A1*, *SCARB1*, and *HSD17B2*) were present in two or more assemblies.

To accurately identify the key genes that may influence porcine litter size, we created a Venn diagram of the 10 most DEGs between high and low litter size samples (Table 3), the 10 most expressed genes in the ovaries of pigs with high litter size (Table 7), the 10 most expressed genes in the ovaries of pigs with low litter size (Table 7), and the 21 genes enriched in the steroid metabolic process and ovarian steroidogenesis of the above-mentioned (S5 Table). The results showed that 11 genes appeared in the two or more sets, including the *CO1*, glutathione peroxidase 3 (*GPX3*), beta-microseminoprotein (*MSMB*), *COX3*, tissue inhibitor of metalloproteinase 1 (*TIMP1*), cytochrome b (*CYTB*), *STAR*, 3-beta hydroxysteroid dehydrogenase (*HSD3B*), *CYP11A1*, *SCARB1*, and hydroxysteroid (17-beta) dehydrogenase 2 (*HSD17B2*) (Fig 3). These 11 genes may be candidates for porcine fecundity and litter size.

**Table 7:** Detailed information of the top 10 most expressed genes in the YH and YL groups.

Group	Gene Name	Readcount_YH	Readcount_YL	log2FoldChange	*P* val	*P* adj	Up/Down(YH/YL)	Interpro Description
YH	*CO1*	1062714.38	183270.39	2.54	6.86E-07	8.59E-05	Up	Cytochrome c oxidase subunit I domain
	*GPX3*	829844.21	63398.00	3.71	2.35E-07	3.43E-05	Up	Glutathione peroxidase 3
	*MSMB*	462726.06	19382.82	4.58	1.90E-18	6.77E-15	Up	Beta-microseminoprotein
	*COX3*	444826.68	99075.58	2.17	1.34E-05	0.001048	Up	Cytochrome c oxidase subunit III domain
	*TIMP1*	407692.96	34203.54	3.58	7.45E-07	9.19E-05	Up	Tissue inhibitor of metalloproteinases-like
	*STAR*	350027.39	29747.05	3.56	1.76E-08	3.57E-06	Up	Steroidogenic acute regulatory protein, animal
	*HSD3B*	256334.52	26353.57	3.28	3.39E-11	1.45E-08	Up	3-beta hydroxysteroid dehydrogenase
	*CYTB*	187523.02	46638.76	2.01	9.15E-05	0.00469	Up	Cytochrome b
	*CYP11A1*	148774.93	9351.70	3.99	2.42E-16	3.04E-13	Up	Cytochrome P450 E class group 1
	*SCARB1*	128598.74	10691.72	3.59	9.40E-14	7.03E-11	Up	Scavenger receptor class B, member 1
YL	*CO1*	1062714.38	183270.39	2.54	6.86E-07	8.59E-05	Up	Cytochrome c oxidase, subunit I domain
	*COX3*	444826.68	99075.58	2.17	1.34E-05	0.001048	Up	Cytochrome c oxidase subunit III domain
	*COL1A2*	25163.69	68810.42	-1.45	0.000946	0.02541	Down	Collagen triple helix repeat
	*GPX3*	829844.21	63398.00	3.71	2.35E-07	3.43E-05	Up	Glutathione peroxidase 3
	*CYTB*	187523.02	46638.76	2.01	9.15E-05	0.00469	Up	Cytochrome b
	*TIMP1*	407692.96	34203.54	3.58	7.45E-07	9.19E-05	Up	Tissue inhibitor of metalloproteinases-like
	*STAR*	350027.39	29747.05	3.56	1.76E-08	3.57E-06	Up	Steroidogenic acute regulatory protein, animal
	*SEPP1*	81114.92	29232.10	1.47	0.001024	0.027035	Up	Selenoprotein P precursor 1
	*HSD3B*	256334.52	26353.57	3.28	3.39E-11	1.45E-08	Up	3-beta hydroxysteroid dehydrogenase
	*MSMB*	462726.06	19382.82	4.58	1.90E-18	6.77E-15	Up	Beta-microseminoprotein

## Discussion

Ovaries are one of the most important animal reproductive organs. They directly mediate ovulation and female hormone secretion, which has a significant impact on the fecundity of mammals [34–36]. Some specific genes relating to animal fecundity have been reported using transcriptome analysis of certain characteristics in ovaries [18, 37], however similar studies on litter size in pigs had not been conducted to date. It is well-known that there is gene expression specificity in different tissues and cells. The ovaries contain a mixture of different tissue, and the expression of candidate genes may differ between them. We strove to ensure that we obtained intact ovaries and ground them completely for the purpose of RNA extraction, to ensure that the RNA-Seq results were representative of the complete porcine ovarian transcriptome. Additionally, the age of the sow is known to greatly affect litter size. In order to minimize this and the effect of parity, we selected six pigs of similar age and parity for RNA-Seq. Two sequencing libraries were constructed from extremely high and low litter size samples. High quality transcriptome data was generated (60 million clean reads; 80% genome mapping rate), which was sufficient for the quantitative analysis of gene expression.

Previous studies of goat ovaries suggested that the most differentially expressed genes identified by RNA-Seq were likely to be important for improving litter size [19]. Some of the top 10 most differentially expressed genes found in our study have been reported to be candidate genes involved in important metabolic processes. For example: the *PCK1* gene has previously been shown to be associated with lipid metabolism in animals [38–41]; the *HSD17B2* gene has been identified as a key regulator of steroid hormone metabolism [42–46]; the *EGR4* gene is a candidate gene for reproductivity in animal ovary and testis [47–50]; and the *RAB33A* gene may affect the risk of developing certain disease, such as tuberculosis [51, 52]. However, while there was an association between *HGD* and meat quality traits reported in Chinese red cattle [53, 54], these gene association studies have mainly concentrated on humans or animal models. Therefore, the role of these genes in pig fecundity requires further investigation.

From our GO and KEGG analysis, we found that the functions of the 10 most DEGs between high and low litter size samples were mainly in the metabolic pathways, ovarian steroidogenesis, citrate cycle (TCA cycle), pyruvate metabolism, and the PPAR signaling pathway. It is accepted that the molecular regulation of animal traits is very complex and the relationship between genes and traits is often that of “one-to-many” or “many-to-one”. The DEGs were not only enriched in reproduction-related pathways but also in those involved with lipid and fatty acid metabolism. This suggests that the genes may be associated with both reproduction and fat metabolism. It is easily appreciated that, in the life of organisms, all physiological processes interact with each other. The pathways mentioned above are, to a greater or lesser degree, involved in the development of follicular cells and oocytes. Consequently, functional studies should be performed with these DGEs in order to identify key candidate genes influencing reproductive traits in swine.

Previous research with gene expression microarrays and QTL mapping analysis, identified 27 candidate genes, co-localizing with QTL for porcine litter size traits, which fulfill biological, positional, and functional criteria [55, 56]. These genes, which include *CYP19A1*, *CYP2E1*, *RBP4*, *MSRB2*, and *SLC16A3*, mainly encode specific proteins, hormones and cytokines. *CYP11A1* (which belongs to cytochrome P450 E class group 1), *RBP4* (retinol-binding protein 4), and *SLC5A10*, *SLC7A11* & *SLC6A20B* (which belong to the solute carrier family) were also identified in our study as DEGs in pigs, suggesting that they may also be relevant to porcine prolificacy (S2 Table). However, apart from these DEGs, the other candidate genes found previously to co-localize with QTL for porcine litter size were not identified in our study. This may be because of inter-species differences, or it may be a consequence of an insufficiently large sample size.

Genes that are highly expressed in reproductive tissues or cells may also indicate that the gene itself is activated, as shown in previous studies [57–59]. Therefore, we identified the 10 most highly expressed genes in the ovaries of high and low litter size pigs, respectively (Table 7). Eight of these genes (*CO1*, *GPX*3, *MSMB*, *COX3*, *TIMP1*, *STAR*, *HSD3B*, and *CYTB*) were upregulated in the high litter size samples. Some of them may be involved in specific reproductive processes. For example, previous research has shown that *MSMB* participates in spermatogenesis [60], and that *HSD3B* and *STAR* regulate specific gonadal development and hormone metabolism pathways [61–64]. The other most highly expressed genes may play other important roles in biological processes. *CO1* and *COX3* genes have been extensively utilized in polymorphic and phylogenetic analysis in some species [65, 66] and *GPX*3 is associated with certain cancers [67, 68].

Furthermore, in order to identify more candidate genes relate to porcine litter size, we emphatically analyzed the 21 candidate genes (S5 Table) that may be associated with ovarian steroid hormone secretion. We showed that 19 of these 21 genes were all upregulated in the ovaries of pigs with high litter size. This suggests that the ovarian steroidogenesis pathway may be activated. These 21 genes mainly contribute to ovarian steroidogenesis and development of follicular cells.

Finally, by comparing the most highly expressed genes in the high and low litter size samples with the 10 most DEGs, and the 21 genes enriched in the steroid metabolic process and ovarian steroidogenesis, we identified a total of 11 candidate genes (*CO1*, *GPX3*, *MSMB*, *COX3*, *TIMP1*, *CYTB*, *STAR*, *HSD3B*, *CYP11A1*, *SCARB1*, and *HSD17B2*) relating to porcine fecundity and litter size. Therefore, the reproductive roles of these 11 genes in pigs should be further investigated in specific ovarian cells (such as theca and granulosa cells) in order to determine their functions both in vitro and in vivo.

## Conclusion

This study screened for DEGs in the ovarian tissues of extremely high and low litter size Yorkshire pigs using RNA-Seq. We identified 897 genes that were upregulated and 346 genes that were downregulated in the high litter size samples. After analyzing the function of these genes, we found 11 DEGs that may be relevant to the prolificacy of pigs. This new information provides a solid foundation for further studies of the molecular mechanisms underlying porcine prolificacy. In the future, biochemical and physiological analyses of these candidate genes will be conducted.

## Supporting Information

S1 Checklist Completed ARRIVE Guidelines Checklist for the reporting of animal data. (PDF)

S1 Table Primer pairs selected for RNA-Seq validation by qPCR. (XLSX)

S2 Table Detailed information of differentially expressed genes. (XLS)

S3 Table Significantly enriched GO terms among differentially expressed genes. (XLS)

S4 Table Significantly enriched KEGG pathways among differentially expressed genes. (XLS)

S5 Table Detailed information of 21 differentially expressed genes enriched in the ovarian steroidogenesis pathway. (XLSX)

## References

[1] Lee DG, Nam J, Kim SW, Kang YM, An HJ, Kim CW, et al Proteomic analysis of reproduction proteins involved in litter size from porcine placenta. Bioscience, biotechnology, and biochemistry. 2015;79(9):1414–21. 10.1080/09168451.2015.1039478 25921448

[2] Kwon WS, Rahman MS, Lee JS, Yoon SJ, Park YJ, Pang MG. Discovery of predictive biomarkers for litter size in boar spermatozoa. Molecular & cellular proteomics: MCP. 2015;14(5):1230–40. 25693803 10.1074/mcp.M114.045369 PMC4424395

[3] Du H, Chen J, Cui J, Wang X, Zhang X. Polymorphisms on SSC15q21-q26 Containing QTL for reproduction in Swine and its association with litter size. Genetics and molecular biology. 2009;32(1):69–74. 10.1590/S1415-47572009000100010 21637648 PMC3032975

[4] Stephens SM, Moley KH. Follicular origins of modern reproductive endocrinology. American journal of physiology Endocrinology and metabolism. 2009;297(6):E1235–6. 10.1152/ajpendo.00575.2009 19933447

[5] Pan Z, Zhang J, Lin F, Ma X, Wang X, Liu H. Expression profiles of key candidate genes involved in steroidogenesis during follicular atresia in the pig ovary. Molecular biology reports. 2012;39(12):10823–32. 10.1007/s11033-012-1976-2 23053978

[6] Munoz G, Ovilo C, Estelle J, Silio L, Fernandez A, Rodriguez C. Association with litter size of new polymorphisms on ESR1 and ESR2 genes in a Chinese-European pig line. Genetics, selection, evolution: GSE. 2007;39(2):195–206. 17306201 10.1186/1297-9686-39-2-195 PMC2682837

[7] Chen KF, Huang LS, Li N, Zhang Q, Luo M, Wu CX. The genetic effect of estrogen receptor(ESR) on litter size traits in pig. Yi chuan xue bao = Acta genetica Sinica. 2000;27(10):853–7. 11192427

[8] Zhao Y, Li N, Xiao L, Cao G, Chen Y, Zhang S, et al FSHB subunit gene is associated with major gene controlling litter size in commercial pig breeds. Science in China Series C, Life sciences / Chinese Academy of Sciences. 1998;41(6):664–8. 10.1007/BF02882910 18726224

[9] Sun YX, Zeng YQ, Tang H, Fan XZ, Chen QM, Li H, et al Relationship of genetic polymorphism of PRLR and RBP4 genes with litter size traits in pig. Yi chuan = Hereditas / Zhongguo yi chuan xue hui bian ji. 2009;31(1):63–8. 19138903 10.3724/sp.j.1005.2009.00063

[10] Spotter A, Muller S, Hamann H, Distl O. Effect of polymorphisms in the genes for LIF and RBP4 on litter size in two German pig lines. Reproduction in domestic animals = Zuchthygiene. 2009;44(1):100–5. 10.1111/j.1439-0531.2007.01004.x 18537906

[11] Ramirez O, Tomas A, Barragan C, Noguera JL, Amills M, Varona L. Pig melatonin receptor 1a (MTNR1A) genotype is associated with seasonal variation of sow litter size. Animal reproduction science. 2009;115(1–4):317–22. 10.1016/j.anireprosci.2008.12.013 19181464

[12] Goluch D, Korwin-Kossakowska A, Prusak B, Pierzchala M, Urbanski P, Michaluk A, et al The study of polymorphism within the promoter region of the osteopontin (OPN) gene in sows. Neuro endocrinology letters. 2009;30(4):525–9. 20010486

[13] Paradis F, Novak S, Murdoch GK, Dyck MK, Dixon WT, Foxcroft GR. Temporal regulation of BMP2, BMP6, BMP15, GDF9, BMPR1A, BMPR1B, BMPR2 and TGFBR1 mRNA expression in the oocyte, granulosa and theca cells of developing preovulatory follicles in the pig. Reproduction. 2009;138(1):115–29. 10.1530/REP-08-0538 19359354

[14] Shimizu T, Miyahayashi Y, Yokoo M, Hoshino Y, Sasada H, Sato E. Molecular cloning of porcine growth differentiation factor 9 (GDF-9) cDNA and its role in early folliculogenesis: direct ovarian injection of GDF-9 gene fragments promotes early folliculogenesis. Reproduction. 2004;128(5):537–43. 15509699 10.1530/rep.1.00224

[15] Rothschild MF, Hu ZL, Jiang Z. Advances in QTL mapping in pigs. International journal of biological sciences. 2007;3(3):192–7. 17384738 10.7150/ijbs.3.192 PMC1802014

[16] Distl O. Mechanisms of regulation of litter size in pigs on the genome level. Reproduction in domestic animals = Zuchthygiene. 2007;42 Suppl 2:10–6. 17688597 10.1111/j.1439-0531.2007.00887.x

[17] Marioni JC, Mason CE, Mane SM, Stephens M, Gilad Y. RNA-seq: an assessment of technical reproducibility and comparison with gene expression arrays. Genome research. 2008;18(9):1509–17. 10.1101/gr.079558.108 18550803 PMC2527709

[18] Lan D, Xiong X, Wei Y, Xu T, Zhong J, Zhi X, et al RNA-Seq analysis of yak ovary: improving yak gene structure information and mining reproduction-related genes. Science China Life sciences. 2014;57(9):925–35. 10.1007/s11427-014-4678-2 24907937

[19] Ling YH, Xiang H, Li YS, Liu Y, Zhang YH, Zhang ZJ, et al Exploring differentially expressed genes in the ovaries of uniparous and multiparous goats using the RNA-Seq (Quantification) method. Gene. 2014;550(1):148–53. 10.1016/j.gene.2014.08.008 25106856

[20] Chen HY, Shen H, Jia B, Zhang YS, Wang XH, Zeng XC. Differential Gene Expression in Ovaries of Qira Black Sheep and Hetian Sheep Using RNA-Seq Technique. PLOS one. 2015;10(3):e0120170 10.1371/journal.pone.0120170 25790350 PMC4366253

[21] Sodhi SS, Song KD, Ghosh M, Sharma N, Lee SJ, Kim JH, et al Comparative transcriptomic analysis by RNA-seq to discern differential expression of genes in liver and muscle tissues of adult Berkshire and Jeju Native Pig. Gene. 2014;546(2):233–42. 10.1016/j.gene.2014.06.005 24910116

[22] Sodhi SS, Park WC, Ghosh M, Kim JN, Sharma N, Shin KY, et al Comparative transcriptomic analysis to identify differentially expressed genes in fat tissue of adult Berkshire and Jeju Native Pig using RNA-seq. Molecular biology reports. 2014;41(9):6305–15. 10.1007/s11033-014-3513-y 25008993

[23] Jung WY, Kwon SG, Son M, Cho ES, Lee Y, Kim JH, et al RNA-Seq approach for genetic improvement of meat quality in pig and evolutionary insight into the substrate specificity of animal carbonyl reductases. PLOS one. 2012;7(9):e42198 10.1371/journal.pone.0042198 22962580 PMC3433470

[24] Wolf J. Heritabilities and genetic correlations for litter size and semen traits in Czech Large White and Landrace pigs. Journal of animal science. 2010;88(9):2893–903. 10.2527/jas.2009-2555 20495118

[25] Arango J, Misztal I, Tsuruta S, Culbertson M, Herring W. Threshold-linear estimation of genetic parameters for farrowing mortality, litter size, and test performance of Large White sows. Journal of animal science. 2005;83(3):499–506. 15705745 10.2527/2005.833499x

[26] Kilkenny C, Browne WJ, Cuthill IC, Emerson M, Altman DG. Improving bioscience research reporting: the ARRIVE guidelines for reporting animal research. PLOS biology. 2010;8(6):e1000412 10.1371/journal.pbio.1000412 20613859 PMC2893951

[27] Kerpedjiev P, Frellsen J, Lindgreen S, Krogh A. Adaptable probabilistic mapping of short reads using position specific scoring matrices. BMC bioinformatics. 2014;15 10.1186/1471-2105-15-15 24717095 PMC4021105

[28] Kim D, Salzberg SL. TopHat-Fusion: an algorithm for discovery of novel fusion transcripts. Genome Biol. 2011;12(8):R72 10.1186/gb-2011-12-8-r72 21835007 PMC3245612

[29] Mortazavi A, Williams BA, Mccue K, Schaeffer L, Wold B. Mapping and quantifying mammalian transcriptomes by RNA-Seq. Nature methods. 2008;5(7):621–8. 10.1038/nmeth.1226 18516045 PMC13303166

[30] Benjamini Y, Drai D, Elmer G, Kafkafi N, Golani I. Controlling the false discovery rate in behavior genetics research. Behav Brain Res. 2001;125(1–2):279–84. 11682119 10.1016/s0166-4328(01)00297-2

[31] Ashburner M, Ball CA, Blake JA, Botstein D, Butler H, Cherry JM, et al Gene Ontology: tool for the unification of biology. Nat Genet. 2000;25(1):25–9. 10802651 10.1038/75556 PMC3037419

[32] Kanehisa M, Araki M, Goto S, Hattori M, Hirakawa M, Itoh M, et al KEGG for linking genomes to life and the environment. Nucleic acids research. 2008;36:D480–D4. 18077471 10.1093/nar/gkm882 PMC2238879

[33] Steibel JP, Poletto R, Coussens PM, Rosa GJM. A powerful and flexible linear mixed model framework for the analysis of relative quantification RT-PCR data. Genomics. 2009;94(2):146–52. 10.1016/j.ygeno.2009.04.008 19422910

[34] Weiner S, Wright KH, Wallach EE. Studies on the function of the denervated rabbit ovary: human chorionic gonadotropin-induced ovulation. Fertility and sterility. 1975;26(4):363–8. 1116631

[35] Paeschke KD. Ovulation preliminaries and ovulation. I. Generative function of the ovary and ascorbic acid metabolism during the ovarian cycle. Fortschritte der Geburtshilfe und Gynakologie. 1970;43:1–58. 4104039

[36] Rojas FJ. Ovulation induction. Effects of ovulation induction with gonadotrophins on the ovary and uterus and their implications for assisted reproduction. Human reproduction. 1995;10(9):2219–24. 8530638 10.1093/oxfordjournals.humrep.a136271

[37] Chen L, Liu K, Zhao Z, Blair HT, Zhang P, Li D, et al Identification of sheep ovary genes potentially associated with off-season reproduction. Journal of genetics and genomics = Yi chuan xue bao. 2012;39(4):181–90. 10.1016/j.jgg.2012.03.002 22546540

[38] Metges CC, Gors S, Lang IS, Hammon HM, Brussow KP, Weitzel JM, et al Low and high dietary protein:carbohydrate ratios during pregnancy affect materno-fetal glucose metabolism in pigs. The Journal of nutrition. 2014;144(2):155–63. 10.3945/jn.113.182691 24353346

[39] Guo F, Zhang Y, Su L, Ahmed AA, Ni Y, Zhao R. Breed-dependent transcriptional regulation of phosphoenolpyruvate carboxylase, cytosolic form, expression in the liver of broiler chickens. Poultry science. 2013;92(10):2737–44. 10.3382/ps.2013-03189 24046422

[40] Duan J, Shao F, Shao Y, Li J, Ling Y, Teng K, et al Androgen inhibits abdominal fat accumulation and negatively regulates the PCK1 gene in male chickens. PLOS one. 2013;8(3):e59636 10.1371/journal.pone.0059636 23544081 PMC3609855

[41] Hsieh CW, Huang C, Bederman I, Yang J, Beidelschies M, Hatzoglou M, et al Function of phosphoenolpyruvate carboxykinase in mammary gland epithelial cells. Journal of lipid research. 2011;52(7):1352–62. 10.1194/jlr.M012666 21504969 PMC3122918

[42] Wang CL, Ying SJ, Wang ZY, Xing HJ, Wang LZ, He DY, et al Molecular cloning and expression of 17beta-hydroxysteroid dehydrogenase type 2 gene in Hu sheep. Molecular biology reports. 2013;40(2):1073–80. 10.1007/s11033-012-2149-z 23096084

[43] Gao H, Yallampalli U, Yallampalli C. Gestational protein restriction reduces expression of Hsd17b2 in rat placental labyrinth. Biology of reproduction. 2012;87(3):68 22837477 10.1095/biolreprod.112.100479 PMC3464908

[44] Shen Z, Peng Z, Sun Y, Vaananen HK, Poutanen M. Overexpression of human hydroxysteroid (17beta) dehydrogenase 2 induces disturbance in skeletal development in young male mice. Journal of bone and mineral research: the official journal of the American Society for Bone and Mineral Research. 2008;23(8):1217–26. 10.1359/jbmr.080322 18348690

[45] Rantakari P, Strauss L, Kiviranta R, Lagerbohm H, Paviala J, Holopainen I, et al Placenta defects and embryonic lethality resulting from disruption of mouse hydroxysteroid (17-beta) dehydrogenase 2 gene. Molecular endocrinology. 2008;22(3):665–75. 18048640 10.1210/me.2007-0257 PMC5419621

[46] Zhongyi S, Rantakari P, Lamminen T, Toppari J, Poutanen M. Transgenic male mice expressing human hydroxysteroid dehydrogenase 2 indicate a role for the enzyme independent of its action on sex steroids. Endocrinology. 2007;148(8):3827–36. 17510238 10.1210/en.2007-0365

[47] Wang J, Zhao Y, Gu K, Yu P, Zhang B, Wang W, et al The novel porcine gene early growth response 4 (Egr4) is differentially expressed in the ovaries of Erhualian and Pietrain pigs. Reproduction, fertility, and development. 2014;26(4):587–98. 10.1071/RD12380 23719176

[48] Hogarth CA, Mitchell D, Small C, Griswold M. EGR4 displays both a cell- and intracellular-specific localization pattern in the developing murine testis. Developmental dynamics: an official publication of the American Association of Anatomists. 2010;239(11):3106–14. 20925118 10.1002/dvdy.22442 PMC3218559

[49] Tourtellotte WG, Nagarajan R, Bartke A, Milbrandt J. Functional compensation by Egr4 in Egr1-dependent luteinizing hormone regulation and Leydig cell steroidogenesis. Molecular and cellular biology. 2000;20(14):5261–8. 10866682 10.1128/mcb.20.14.5261-5268.2000 PMC85975

[50] Tourtellotte WG, Nagarajan R, Auyeung A, Mueller C, Milbrandt J. Infertility associated with incomplete spermatogenic arrest and oligozoospermia in Egr4-deficient mice. Development. 1999;126(22):5061–71. 10529423 10.1242/dev.126.22.5061

[51] Nakazawa H, Sada T, Toriyama M, Tago K, Sugiura T, Fukuda M, et al Rab33a mediates anterograde vesicular transport for membrane exocytosis and axon outgrowth. The Journal of neuroscience: the official journal of the Society for Neuroscience. 2012;32(37):12712–25. 22972995 10.1523/JNEUROSCI.0989-12.2012 PMC6703789

[52] Jacobsen M, Repsilber D, Gutschmidt A, Neher A, Feldmann K, Mollenkopf HJ, et al Ras-associated small GTPase 33A, a novel T cell factor, is down-regulated in patients with tuberculosis. The Journal of infectious diseases. 2005;192(7):1211–8. 16136464 10.1086/444428

[53] Zhou GL, Cao Y, Li M, Zhang LC, Yu YS, Jin HG. Meat quality and carcass traits in relation to HGD-BstXI and HGD-HaeIII PCR-RFLP polymorphism in Chinese red cattle. Meat Sci. 2010;85(2):270–3. 10.1016/j.meatsci.2010.01.011 20374897

[54] Zhou G, Dudgeon C, Li M, Cao Y, Zhang L, Jin H. Molecular cloning of the HGD gene and association of SNPs with meat quality traits in Chinese red cattle. Molecular biology reports. 2010;37(1):603–11. 10.1007/s11033-009-9860-4 19816789

[55] Fernandez-Rodriguez A, Munoz M, Fernandez A, Pena RN, Tomas A, Noguera JL, et al Differential gene expression in ovaries of pregnant pigs with high and low prolificacy levels and identification of candidate genes for litter size. Biology of reproduction. 2011;84(2):299–307. 10.1095/biolreprod.110.085589 20926806

[56] Noguera JL, Rodriguez C, Varona L, Tomas A, Munoz G, Ramirez O, et al A bi-dimensional genome scan for prolificacy traits in pigs shows the existence of multiple epistatic QTL. BMC genomics. 2009;10:636 10.1186/1471-2164-10-636 20040109 PMC2812473

[57] Xia J, Yuan J, Xin L, Zhang Y, Kong S, Chen Y, et al Transcriptome analysis on the inflammatory cell infiltration of nonalcoholic steatohepatitis in bama minipigs induced by a long-term high-fat, high-sucrose diet. PLOS one. 2014;9(11):e113724 10.1371/journal.pone.0113724 25415189 PMC4240652

[58] Wang X, Zhou G, Xu X, Geng R, Zhou J, Yang Y, et al Transcriptome profile analysis of adipose tissues from fat and short-tailed sheep. Gene. 2014;549(2):252–7. 10.1016/j.gene.2014.07.072 25088569

[59] Gan L, Xie L, Zuo F, Xiang Z, He N. Transcriptomic analysis of Rongchang pig brains and livers. Gene. 2015;560(1):96–106. 10.1016/j.gene.2015.01.051 25637719

[60] Anahi Franchi N, Avendano C, Molina RI, Tissera AD, Maldonado CA, Oehninger S, et al beta-Microseminoprotein in human spermatozoa and its potential role in male fertility. Reproduction. 2008;136(2):157–66. 10.1530/REP-08-0032 18469041

[61] Sechman A, Antos P, Katarzynska D, Grzegorzewska A, Wojtysiak D, Hrabia A. Effects of 2,3,7,8-tetrachlorodibenzo-p-dioxin on secretion of steroids and STAR, HSD3B and CYP19A1 mRNA expression in chicken ovarian follicles. Toxicology letters. 2014;225(2):264–74. 10.1016/j.toxlet.2013.12.021 24398026

[62] Nakamoto M, Fukasawa M, Tanaka S, Shimamori K, Suzuki A, Matsuda M, et al Expression of 3beta-hydroxysteroid dehydrogenase (hsd3b), star and ad4bp/sf-1 during gonadal development in medaka (Oryzias latipes). General and comparative endocrinology. 2012;176(2):222–30. 10.1016/j.ygcen.2012.01.019 22330050

[63] Tosser-Klopp G, Mulsant P, Yerle M. Regional localisations of VIM, HSD3b, ACTA1 and PGM1 in pigs. Animal genetics. 1998;29(1):23–6. 9682444 10.1046/j.1365-2052.1998.00226.x

[64] Sechman A, Pawlowska K, Hrabia A. Effect of 3,3',5-triiodothyronine and 3,5-diiodothyronine on progesterone production, cAMP synthesis, and mRNA expression of STAR, CYP11A1, and HSD3B genes in granulosa layer of chicken preovulatory follicles. Domestic animal endocrinology. 2011;41(3):137–49. 10.1016/j.domaniend.2011.05.007 21798688

[65] Moazeni M, Sharifiyazdi H, Izadpanah A. Characterization of Fasciola hepatica genotypes from cattle and sheep in Iran using cytochrome C oxidase gene (CO1). Parasitology research. 2012;110(6):2379–84. 10.1007/s00436-011-2774-9 22186976

[66] Tian Z, Liu G, Yin H, Luo J, Guan G, Xie J, et al Cytochrome c oxidase subunit III (COX3) gene, an informative marker for phylogenetic analysis and differentiation of Babesia species in China. Infection, genetics and evolution: journal of molecular epidemiology and evolutionary genetics in infectious diseases. 2013;18:13–7. 10.1016/j.meegid.2013.04.002 23619098

[67] Zhao H, Li J, Li X, Han C, Zhang Y, Zheng L, et al Silencing GPX3 Expression Promotes Tumor Metastasis in Human Thyroid Cancer. Current protein & peptide science. 2015;16(4):316–21. 25929866 10.2174/138920371604150429154840

[68] Cao S, Yan B, Lu Y, Zhang G, Li J, Zhai W, et al Methylation of promoter and expression silencing of GPX3 gene in hepatocellular carcinoma tissue. Clinics and research in hepatology and gastroenterology. 2015;39(2):198–204. 10.1016/j.clinre.2014.09.003 25445749

